# Prevalence of Xpert MTB/RIF Ultra Trace Call Results and Associated Risk Factors During Active Tuberculosis Case Finding in Viet Nam: A Programmatic Evaluation

**DOI:** 10.3390/diagnostics15081006

**Published:** 2025-04-15

**Authors:** Luong Van Dinh, Khoa Tu Tran, Andrew James Codlin, Luan Nguyen Quang Vo, Nga Thuy Thi Nguyen, Lan Phuong Nguyen, Rachel Forse, Han Thi Nguyen, Thi Minh Ha Dang, Lan Huu Nguyen, Hoa Binh Nguyen, Jacob Creswell

**Affiliations:** 1 National Lung Hospital, Ha Noi 100000, Vietnam; dinhvanluong66@gmail.com (L.V.D.); nguyenbinhhoatb@yahoo.com (H.B.N.); 2 Friends for International TB Relief, Ha Noi 100000, Vietnam; tukhoatran95@gmail.com (K.T.T.); nga.nguyen@tbhelp.org (N.T.T.N.); rachel.forse@tbhelp.org (R.F.); han.nguyen@tbhelp.org (H.T.N.); 3 Department of Global Public Health, WHO Collaboration Centre on Tuberculosis and Social Medicine, Karolinska Institutet, Solna, 17176 Stockholm, Sweden; 4 IRD VN Social Enterprise Ltd., Ho Chi Minh City 700000, Vietnam; lan.nguyen@ird.vn; 5 Pham Ngoc Thach Hospital, Ho Chi Minh City 700000, Vietnam; hadtm2023@gmail.com (T.M.H.D.); nguyenhuulan1965@gmail.com (L.H.N.); 6 Stop TB Partnership, 1218 Geneva, Switzerland; jacobc@stoptb.org

**Keywords:** trace call, Xpert MTB/RIF Ultra, mobile CXR screening, TB, active TB case-finding, chest X-ray, tuberculosis

## Abstract

**Background:** The Xpert MTB/RIF Ultra assay (Ultra) is a second-generation molecular diagnostic test for tuberculosis (TB). The “Trace Call” result was added as a semi-quantitative category for extremely low bacillary loads. The prevalence and interpretation of Trace Call results remains insufficiently elucidated in the context of community-based active case finding (ACF). **Methods:** We organized 56 days of mobile chest X-ray (CXR) screening events in Ho Chi Minh City, Viet Nam, between October 2020 and March 2021. Participants were screened verbally and by CXR and tested by Ultra, if eligible. Persons with a Trace Call were re-tested on Ultra per national guidelines. qXRv3 computer-aided detection software was used for post hoc quality control of CXR interpretation. We calculated descriptive statistics and fitted mixed-effect multivariate regression models to identify factors associated with Trace Call results and confirmatory diagnosis. **Results:** A total of 16,698 people were screened by CXR to detect 185 Ultra-positive participants, including 142 persons with a confirmed TB diagnosis. Among Ultra-positive participants, 38.4% (71/185) had Trace Call results. Of these, 85.9% (61/71) were re-tested, and 45.9% (28/61) were bacteriologically-confirmed, comprising 19.7% (28/142) of the final number of confirmed diagnoses. Having a low qXR abnormality score (<0.5) (aOR = 4.97; 95%CI: [1.88, 13.14]; *p* = 0.001) and a history of TB within 5 recent years (aOR = 3.53; 95%CI: [1.69, 7.35]; *p* = 0.001) were associated with an initial Trace Call. **Conclusions:** The Trace Call can improve ACF detection, particularly in earlier stages of disease with limited pulmonary deterioration. However, participants with a history of TB had higher rates of Trace Call, reinforcing the need to interpret test results in this group with caution.

## 1. Introduction

Tuberculosis (TB) remains a leading cause of death worldwide, despite being both preventable and curable [1]. Low TB treatment coverage, which was exacerbated by the COVID-19 pandemic, is the driver of continued transmission, morbidity, and mortality. One strategy for improving the detection and treatment of TB is active case finding (ACF) outside of health facilities [2]. Such initiatives provide opportunities for individuals who may lack access to specialized healthcare facilities or those in the early stages of active TB, when symptoms may not be apparent or are mild, to receive a timely diagnosis. To ensure the success of these resource-intensive initiatives and provide the highest quality of care to individuals screened through ACF, it is crucial to employ highly sensitive diagnostic tools.

The Xpert MTB/RIF assay (Xpert; Cepheid; Sunnyvale, CA, USA) is a rapid molecular diagnostic test initially recommended by the WHO in 2011, which could detect TB and rifampicin resistance in less than two hours based on genetic material of TB present in a sputum sample [3]. However, Xpert’s diagnostic performance in smear-negative individuals and people living with HIV, while higher than microscopy, is suboptimal. Specifically, sensitivity was lower, and the risk of false positive rifampicin-resistance results was higher in these groups [4,5]. To address these challenges, the successor to this assay, Xpert MTB/RIF Ultra (Ultra), was developed [6]. Ultra has shown improved sensitivity in the lowest bacillary burden. To classify this lower level of detection, the assay has expanded the four semi-quantitative categories of the original Xpert assay (high, medium, low, and very low) by adding a fifth category called “Trace Call” [7]. This Trace Call indicates the presence of a minimum level of bacilli but is unable to determine rifampicin resistance and in clinical practice may present challenges of interpretations [8,9]. Xpert was completely discontinued in 2023, and all new tests used are Ultra assays. However, the improvement comes at the cost of lower specificity, higher false positives, and potential unnecessary treatment [8]. This is because Ultra can detect genetic material of non-replicating or non-viable bacilli that may still be present in patients from a prior episode of TB. Therefore, WHO has recommended a specific approach to interpret a Trace Call, including conducting a second Ultra assay unless exempted, e.g., for HIV-positive individuals, children, and persons with presumptive TB meningitis and other severe forms. This approach is particularly important for high-burden TB countries given the large number of people with a recent history of TB [10].

Viet Nam is one of the countries with a high burden of TB. In 2023, it reported an estimated number of more than 182,000 persons with incident TB, placing it among countries with the highest burden of TB [11]. The 2014 national strategy for TB set the vision to reduce TB prevalence to below 20/100,000 in Viet Nam, and Resolution 20-NQ/TW in 2017 targets the elimination of TB by 2030 [12,13]. In response, the National TB Control Programme (NTP) piloted the Xpert MTB/RIF assay for first-line diagnostic testing for individuals with signs of TB on chest X-ray (CXR), locally named the Double-X algorithm, in 2017 and instituted this algorithm under routine care in 2020. That same year, guidelines were published to provide guidance on the follow-up diagnostic steps for Trace Call results. These guidelines were primarily based on WHO recommendations [10]. According to national guidelines, individuals who receive an initial Trace Call and do not meet exemption criteria are required to undergo a second confirmatory Ultra test. Clinical consultation may be necessary afterward to assess the individual’s specific circumstances and determine the necessity of treatment [14,15].

While Ultra is an accurate assay for TB diagnosis, the trade-off between sensitivity and specificity remains insufficiently investigated, especially among persons with a history of TB. Moreover, the role of repeat testing of a fresh sputum sample in persons with an initial Trace Call needs further evaluation to determine if it improves specificity in the context of ACF [10,16,17]. The inclusion of the Trace Call category may help detect more persons with TB, especially in TB endemic settings and within high-prevalence health facilities, and reduce misdiagnosis resulting from low TB bacterial load [9,18,19]. This has the potential to enhance the yield and efficiency of ACF initiatives. While prior studies have assessed the performance of Ultra in ACF, evidence specifically focusing on the role of Trace Call results in ACF remains limited [17,20]. This study aims to contribute to this knowledge base by evaluating the prevalence of Trace Call results during TB ACF in Viet Nam, exploring the characteristics of Trace Call results, and identifying associated risk factors in a high TB burden setting.

## 2. Materials and Methods

### 2.1. Design

This was a cross-sectional analysis of diagnostic test results using the Xpert MTB/RIF Ultra assay during a community-based ACF intervention for TB in a routine programmatic setting.

### 2.2. Setting

Between October 2020 and March 2021, 56 days of community-based, mobile CXR screening events for TB were conducted across the following three districts of Ho Chi Minh City (HCMC): District 8, Binh Chanh and Go Vap. These intervention districts had a combined population of 1.8 million and notified 2663 people treated for TB in 2019 (case notification rate of 164 per 100,000 population).

**Figure 1:**
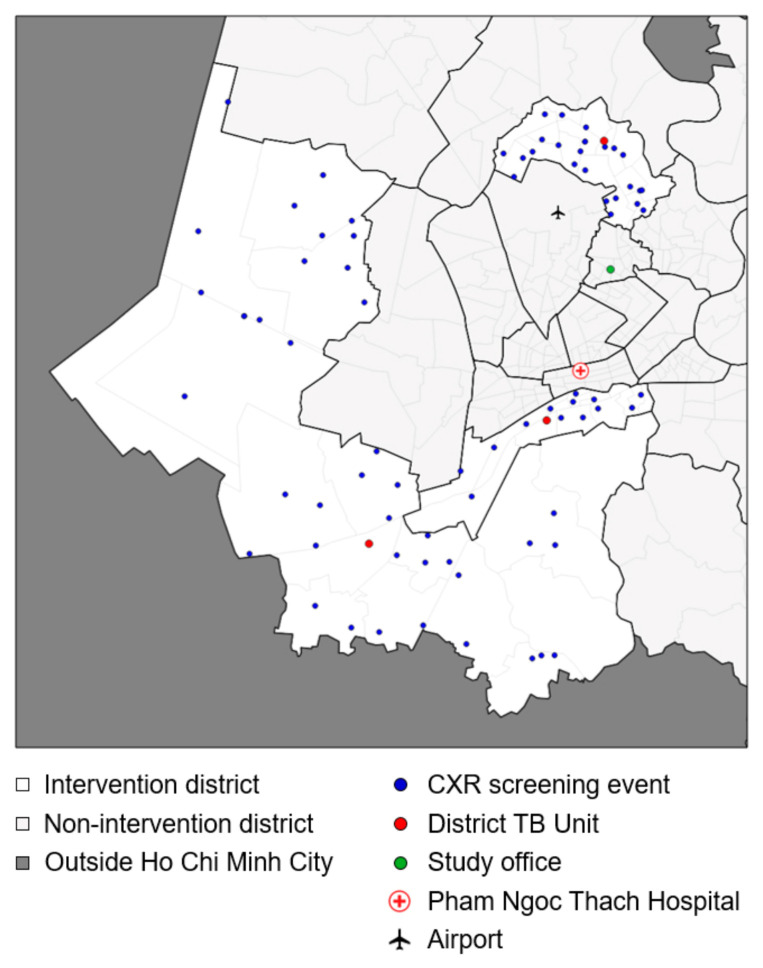
Map of locations of mobile CXR screening events.

The locations of the mobile CXR events (Figure 1) were carefully selected to avail screening within a reasonable distance for dense population clusters across all districts. This ensured that individuals at risk of TB had the opportunity to undergo screening at an event conveniently located near their place of residence. Sites of the screening events were jointly selected in coordination with district TB, health, and administrative authorities approximately two weeks before the implementation. Screening events were usually held at Commune Health Stations, local government offices, stadiums, and schools to have open spaces for gathering participants and stable supply of water, electricity, and sanitation facilities.

### 2.3. Target Population

The ACF events targeted urban priority populations for TB as previously defined elsewhere [21]. These included household contacts, individuals aged 55 years and above, economic migrants, individuals living with HIV or diabetes, residents of boarding home communities and low-income housing, and those exhibiting TB symptoms.

### 2.4. Mobilization

Participant mobilization mirrored previously reported approaches [22]. Briefly, to maximize participation, we collaborated with district and commune public health staff, socio-political organizations such as the Women’s Union, Retirement Association, Red Cross, and available networks of community health workers. Their role was to raise awareness about TB and mobilize community members in the catchment area to attend the upcoming CXR screening events. In addition, personalized letters of invitation were distributed by each commune’s administrative government, notices were posted on community announcement boards, and banners hung in prominent areas. Lastly, CXR screening events were primarily scheduled on weekends and occasionally on Fridays, to improve access for individuals unable to miss work during the week. Screening events were occasionally moved to a new location at midday to maximize mobilization and reach.

### 2.5. Screening and Testing

At the screening events, participants were first verbally screened for TB symptoms using a questionnaire loaded onto a bespoke mHealth app (ACIS, Viet Nam NTP) and then were screened by CXR, irrespective of their symptom presentation. We employed vans with digital radiography systems mounted inside the cargo hold. Digital CXR images were read in real-time by certificated radiologists, and persons with an abnormal CXR were indicated for testing. Per discretion of the attending physician, testing could also be indicated “off-algorithm” based on clinical suspicion despite the absence of TB-related abnormalities on CXR. There was a programmatic difference in the interpretation of CXRs in Go Vap district compared to the other two sites. As Go Vap faced a local Xpert cartridge shortage, CXR images with abnormalities that may not be related to TB were not indicated for testing. At the end of each day, specimens were transported to the nearest laboratory located at the District TB Unit for testing with the Ultra. Xpert tests were conducted by well-trained laboratory staff, with regular External Quality Assurance monitoring conducted by the regional reference laboratory at Pham Ngoc Thach Hospital (PNTH).

Participants with Ultra-positive test results were linked to appropriate TB care at either their nearest District TB Unit for drug-sensitive TB or to the Programmatic Management of Drug-Resistant TB unit at PNTH for further evaluation and treatment in the event of a rifampicin-resistant test result. If the Ultra result was a Trace Call, public health staff contacted the participant to collect a second sputum specimen for confirmatory testing. If a participant’s second Ultra was positive, including a second Trace Call result, they were linked to TB treatment. In the event of discordant results, participants were eligible for a medical consultation and clinical diagnosis. Persons with TB were linked to care in accordance with national treatment guidelines [15].

### 2.6. Statistical Analyses

Pseudonymized data were abstracted from the ACIS mHealth system for this analysis. In addition, CXR DICOM files from the screening events were processed using qXR computer-aided detection (CAD) software (v3; Qure.ai; Mumbai, India). These retrospectively obtained CAD scores were used only for this analysis, using the manufacture-recommended threshold of 0.50; all decisions about sputum testing were made by the on-site radiologists at the screening events [23].

A cascade of care was populated, showing yields from symptom screening to the detection and confirmation of Trace Call results, disaggregated by intervention district. Participants with an initial Ultra-positive test result were bifurcated into those with a Trace Call result and all remaining Ultra-positive participants. Descriptive statistics for the two cohorts were calculated across eight demographic and clinical variables: gender, age group, positive on the WHO 4-symptom screen (cough, fever, weight loss, and/or night sweats), presence of a comorbidity (HIV, diabetes, and/or immune disease), past history of TB, contact of TB patient, qXR score, and the district in which they were screened.

We calculated adjusted odds ratios (aORs) and *p*-values for all demographic and clinical covariates by fitting a mixed-effect logistic regression onto the data, with district as the random effect. Hypothesis tests were 2-sided, and a *p* < 0.05 was considered statistically significant. Analyses were conducted in Stata (v14.2; StataCorp LLC; College Station, TX, USA).

## 3. Results

We implemented 56 community-based CXR screening events in 79 unique locations (Table 1). Overall, 17,020 participants attended the events, and 16,698 (98.1%) participants consented to be screened by CXR (average of 298 participants per day), resulting in the detection of 1942 (11.6%) people with abnormal CXR results. A total of 1729 (10.4%) Ultra tests were conducted, including 1482 following an abnormal CXR and 247 additional off-algorithm tests. This testing yielded 185 participants with Ultra-positive results. Among these, there were 71 (38.4%) Trace Call results. A second sputum sample was collected from 61 (85.9%) of the participants with initial Trace Call results, of which 28 (45.9%) were positive upon retesting, including five (17.9%) second Trace Call results. These 28 bacteriologic confirmations accounted for 19.7% of the 142 persons with a confirmed TB diagnosis, representing an overall TB detection rate of 850 per 100,000. Of these, 130 (91.5%) persons were linked to appropriate treatment.

There were 24 days of community screening in both Binh Chanh and Go Vap, and 8 days in District 8 to achieve 6139, 6960 and 3599 CXR screens, respectively. The CXR abnormality rate was lower in Go Vap (6.6%) compared to Binh Chanh (15.6%) and District 8 (14.6%) given the exclusion of non-TB/other abnormalities on CXR. These CXR screens resulted in 774 (12.6%), 545 (15.1%), and 410 (5.9%) tests in Binh Chanh, District 8, and Go Vap, respectively, to yield 76 (9.8%), 65 (11.9%), and 44 (10.7%) respective Ultra-positive results. About 55.6% of retesting in Binh Chanh yielded positive results compared to 44.0% in District 8 and only 22.2% in Go Vap.

Of 185 participants with an initial positive Ultra test result, 19.5% were female, 57.3% were aged 40–64 years, and 38.4% were 65+ years (Table 2). Moreover, 23.8%, 35.1%, and 41.1% were located in District 8, Binh Chanh, and Go Vap, respectively. In terms of clinical characteristics, 57.3% were asymptomatic, 85.4% reported no comorbidities, 31.4% had a history of TB within the past five years, 9.2% were a contact of a person with TB, and 13.5% had a qXR score of <0.5.

Of the 61 participants with an initial Trace Call who were reached and agreed to be retested, 26.2% were female, 49.2% were aged 40–64 years, and 45.9% were 65+ years. Of these retested participants, 14.8% resided in District 8, 41.0% in Binh Chanh, and 44.3% in Go Vap. Similar to the initial Ultra-positive cohort, 57.4% of participants in this group were asymptomatic, and 86.9% reported no comorbidities. However, a larger proportion (44.3%) reported an episode of TB in the past 5 years, was a contact of someone with TB (11.5%), and had a qXR score of <0.5 (27.9%). There were no statistically significant differences in characteristics between participants in this cohort.

Among Ultra-positive participants, there were two covariates significantly associated with an initial Trace Call (Table 3). Specifically, participants had a significantly higher risk of a Trace Call if they had a history of TB within the past five years (aOR = 3.53; 95%CI: [1.69, 7.35]; *p* = 0.001) or received a qXR score <0.5 (aOR = 4.97; 95%CI: [1.88, 13.14]; *p* = 0.001). No statistically significant differences were identified in participants with confirmatory diagnosis upon retesting.

## 4. Discussion

Our study found that the rate of Trace Call results was high, and the contribution to detection yield was substantial in our ACF approach in a high TB burden setting. Specifically, Trace Call results accounted for 38.4% (71/185) of initial Ultra-positive diagnoses. Following retesting, these initial Trace Call results contributed 19.7% (28/142) to the total confirmed TB yield. We further found that a history of treated TB within the past five years and a qXR score < 0.5 were associated with higher rates of Trace Call.

The Ultra assay has been used for ACF initiatives for several years, generating ample evidence on its positive impact on detecting persons with TB through these initiatives. The TB detection rate in our study using Ultra (850 per 100,000) surpassed the unadjusted detection/prevalence rate in the same ACF initiative in prior years that used the first-generation Xpert (741 per 100,000) by 14.7% [24]. Notably, the yield from ACF using Ultra was five times higher than the contemporaneous case notification rate of the three districts of 164 per 100,000. Our results were concordant with other ACF initiatives using Ultra. For example, a small study from Mozambique showed that using Ultra increased yield from household contact investigation by +74.1%, from 2.7% to 4.8%, compared to Xpert [17]. A Zambian study among presumptive TB patients in a high TB–HIV coinfection setting reported that 32.2% (37/114) of Ultra-positive results were Trace Call [9]. These findings highlight that using Ultra with the new Trace Call result for ACF outperforms Xpert and can detect more persons with TB.

It is well understood that ACF contributes to early detection and treatment [25]. Our study found a high rate of initial Trace Call results in persons with a qXR score of <0.5. This may suggest that these individuals may have been in early stages of disease progression with a low bacterial load and lower rate of pulmonary deterioration. Since chronic pulmonary function impairment can be caused by pulmonary TB, early detection is crucial for patients to receive TB treatment promptly, which has been has been linked to improved treatment outcomes and may help to preserve lung function to avert post-TB lung disease [26,27]. However, the association between artificial intelligence (AI) abnormality scores, disease severity, treatment outcomes and prevention of chronic post-TB sequelae remain knowledge gaps.

Beyond individual benefits, early detection and intervention reduce the risk of TB transmission and consequently lower prevalence in communities burdened by the disease [2,28]. However, the effectiveness of using Ultra to detect early TB disease in high-risk communities also may depend on the approach of ACF. While population-wide screening using Xpert has proven effective to reduce TB prevalence, political commitment remains insufficient to mobilize the investment needed for application of this ACF strategy at scale [29]. To mitigate the cost implications, community-based ACF initiatives often rely on CXR to indicate testing only in conjunction with abnormal radiographic findings suggestive of TB, commonly assessed by a human radiographer. This is particularly important for persons with asymptomatic TB that is exclusively detected by CXR [30,31]. Thus, CXR has reemerged as a critical tool in the fight to end TB and as a key gatekeeper for molecular testing. This approach has recently benefitted from the introduction of CAD software, which is expected to optimize processes and lower costs related to screening and diagnosis, but requires further studies to ascertain the veracity of these claims [23,32].

Even with the use of CXR and CAD software, heterogeneities in mobilization and uncertainties in the implementation environment often introduce variability in participation rates and target populations that diminish TB prevalence and yield in community-based ACF, and subsequently render mass testing prohibitively expensive. Hence, recent work has emerged assessing specimen pooling for Ultra testing [33]. A Laotian study compared individual and pooled samples tested with Xpert and Ultra assays, revealing higher yields with Ultra in both cases. Specifically, the yields were 8.5% (37/436) vs. 6.7% (29/436) when using individual samples and 29.4% (32/109 pools) vs. 22.9% (25/109 pools) when using pooled samples [20]. A natural transition would be the combination of pooling with CAD as an approach to achieve both broad-scale testing in a cost-effective manner as well as higher TB detection yields in early stages of disease progression [34].

While seeking ways to optimize yields through Ultra, our results also suggest interpreting Trace Call results with caution. Out of the initial 71 participants with Trace Call results, 40 had a recent history of TB. As such, our risk analysis confirmed that genetic remnants from a recent episode of TB could result in a Trace Call result. Moreover, considering this, we anticipated a low positive retest rate in this group, with the majority of positive results being Trace Call. However, our post hoc analysis revealed no association between individuals with a history of TB and TB confirmation in retesting. Only 5 of the 28 confirmed cases had a second Trace Call. These findings indicate that regardless of TB history, the proportion of TB confirmation does not differ between these groups. This result was concordant with the Zambian study that also did not detect a difference between patients with and without a history of TB [9]. Thus, we recommend conducting further studies that include culture testing to gain a clearer understanding of the nature of a Trace Call in people with a recent history of TB.

A strength of this study was the existence of standard procedures and routine implementation of large-scale ACF initiatives under programmatic conditions in our high TB burden country setting, into which we were able to seamlessly insert Ultra to evaluate the prevalence and associated risk factors for a Trace Call. This controlled for implementation risks and enabled a high-level comparison with past results using first-generation Xpert assays. Another strength was the inclusion of a retrospectively-obtained CAD score for a generalizable risk factor associated with Trace Call results. This may warrant future studies designed and powered to investigate initial and follow-up Ultra testing results stratified by AI score, lung involvement, and disease staging. Conversely, our study was limited by its cross-sectional nature, lack of culture testing, and our inability to conduct follow-up on health outcomes for individuals who were lost to follow-up along each step of the care cascade, particularly among those that did not receive confirmation on a second Ultra test. Additionally, the sample size of individuals diagnosed with Trace Call was limited. The generalizability of these results is unclear since all screenings were performed in one province within a relatively compact time period before the COVID-19 pandemic. Lastly, the study was implemented in a programmatic setting. During the implementation of the study, there was a shortage of Xpert cartridges in Go Vap district, and eligibility for Xpert testing was more restricted, which was the reason for the lower abnormality rate in that district.

## 5. Conclusions

With its lower limits of detection, the Trace Call of the Ultra assay can improve ACF yields, particularly among people with early stages of the disease. However, further research is needed to account for the effects of previous episodes of TB, assess associations with AI score, compare Trace Call risk factors on ACF versus routine case finding, and determine relevant implications for patient care and overtreatment.

## Figures and Tables

**Table 1:** TB care cascade and Trace Call results by district.

	Binh Chanh		District 8		Go Vap		Total	
	*N*	% ^1^	*N*	% ^1^	*N*	% ^1^	*N*	% ^1^
Days of community screening	24		8		24		56	
Unique ACF event locations	40		14		25		79	
Verbally screened	6212		3661		7147		17,020	
Screened by CXR	6139	98.8%	3599	98.3%	6960	97.4%	16,698	98.1%
Abnormal CXR	958	15.6%	527	14.6%	457	6.6%	1942	11.6%
Tested with Ultra (total)	774	12.6%	545	15.1%	410	5.9%	1729	10.4%
Tested with Ultra after abnormal CXR	693	89.5%	387	71.0%	402	98.0%	1482	85.7%
Tested with Ultra without abnormal CXR	81	10.5%	158	29.0%	8	2.0%	247	14.3%
Ultra-positive test results ^2^	76	9.8%	65	11.9%	44	10.7%	185	10.7%
Very Low, Low, Medium or High test results	46	60.5%	35	53.8%	33	75.0%	114	61.6%
Trace Call test results	30	39.5%	30	46.2%	11	25.0%	71	38.4%
Retesting of Trace Call test results	27	90.0%	25	83.3%	9	81.8%	61	85.9%
Ultra-positive retest results ^2^	15	55.6%	11	44.0%	2	22.2%	28	45.9%
Ultra Trace Call retest results	2	13.3%	2	18.2%	1	50.0%	5	17.9%
Total confirmed TB diagnoses	61	1.0%	46	1.3%	35	0.5%	142	0.9%

CXR = Chest X-ray; TB = Tuberculosis. ^1^ All percentages are calculated as a fraction of the prior super-indicator (i.e., with less indentation) with the exception of the percentage for Total confirmed TB diagnoses, which is calculated as a fraction of the total number of individuals screened by CXR. ^2^ Ultra-positive = High, Medium, Low, Very Low, and Trace Call.

**Table 2:** Participant characteristics.

	Initial Ultra-Positive							Retest						
	Total (*N* = 185)		Positive ^1^ (*n* = 114)		Trace Call (*n* = 71)		*p*-Value ^2^	Total (*N* = 61)		Positive ^3^ (*n* = 28)		Negative (*n* = 33)		*p*-Value ^2^
	*N*	%	*N*	%	*n*	%		*N*	%	*n*	%	*n*	%	
Sex														
Male	149	80.5%	95	83.3%	54	76.1%	0.224	45	73.8%	21	75.0%	24	72.7%	0.841
Female	36	19.5%	19	16.7%	17	23.9%		16	26.2%	7	25.0%	9	27.3%	
Age														
15–39 years	8	4.3%	4	3.5%	4	5.6%	0.479	3	4.9%	1	3.6%	2	6.1%	1.000
40–64 years	106	57.3%	69	60.5%	37	52.1%		30	49.2%	14	50.0%	16	48.5%	
≥65 years	71	38.4%	41	36.0%	30	42.3%		28	45.9%	13	46.4%	15	45.5%	
District														
District 8	44	23.8%	33	29.0%	11	15.5%	0.081	9	14.8%	2	7.1%	7	21.2%	0.250
Binh Chanh	65	35.1%	35	30.7%	30	42.3%		25	41.0%	11	39.3%	14	42.4%	
Go Vap	76	41.1%	46	40.4%	30	42.3%		27	44.3%	15	53.6%	12	36.4%	
W4SS														
No	106	57.3%	63	55.3%	43	60.6%	0.478	35	57.4%	15	53.6%	20	60.6%	0.580
Yes	79	42.7%	51	44.7%	28	39.4%		26	42.6%	13	46.4%	13	39.4%	
Any comorbidity ^4^														
No	158	85.4%	97	85.1%	61	85.9%	0.877	53	86.9%	24	85.7%	29	87.9%	1.000
Yes	27	14.6%	17	14.9%	10	14.1%		8	13.1%	4	14.3%	4	12.1%	
History of TB														
No	105	56.8%	74	64.9%	31	43.7%	**0.011**	27	44.3%	13	46.4%	14	42.4%	0.818
0–5 years	58	31.4%	27	23.7%	31	43.7%		27	44.3%	11	39.3%	16	48.5%	
>5 years	22	11.9%	13	11.4%	9	12.7%		7	11.5%	4	14.3%	3	9.1%	
TB contact														
No	168	90.8%	104	91.2%	64	90.1%	0.803	54	88.5%	25	89.3%	29	87.9%	1.000
Yes	17	9.2%	10	8.8%	7	9.9%		7	11.5%	3	10.7%	4	12.1%	
qXR score														
≥0.50	160	86.5%	106	93.0%	54	76.1%	**0.001**	44	72.1%	20	71.4%	24	72.7%	0.910
<0.50	25	13.5%	8	7.0%	17	23.9%		17	27.9%	8	28.6%	9	27.3%	

TB = Tuberculosis; W4SS = WHO 4-symptom screen (cough, fever, weight loss, night sweats); ^1^ Other positive = High, Medium, Low, Very Low MTB burdens. ^2^ Chi-squared/Fisher’s Exact Test. *p*-Values < 0.05 were bolded to denote a significant difference. ^3^ TB confirmation: positive second Ultra result; No TB confirmation: negative second Ultra result. ^4^ Includes self-reported HIV, diabetes, and/or other illness at the time of screening.

**Table 3:** Risk factors associated with initial Trace Call and confirmatory TB diagnosis.

	Initial Ultra		Retest	
	aOR (95% CI)	*p*-Value ^1^	aOR (95% CI)	*p*-Value ^1^
**Sex**				
Male	ref		ref	
Female	1.44 [0.62, 3.31]	0.396	1.27 [0.35, 4.65]	0.713
**Age**				
15–54 years	ref		ref	
55–74 years	0.72 [0.14, 3.66]	0.694	0.59 [0.04, 9.27]	0.706
≥75 years	0.99 [0.18, 5.26]	0.987	0.63 [0.04, 11.35]	0.757
**W4SS**				
No	ref		ref	
Yes	0.85 [0.44, 1.64]	0.632	0.73 [0.25, 2.13]	0.563
**Any comorbidity**				
No	ref		ref	
Yes	0.86 [0.33, 2.20]	0.750	0.94 [0.17, 5.04]	0.942
**History of TB**				
No	ref		ref	
0–5 years	3.53 [1.69, 7.35]	**0.001**	1.32 [0.40, 4.39]	0.646
>5 years	2.31 [0.84, 6.39]	0.106	0.76 [0.11, 5.44]	0.787
**TB contact**				
No	ref		ref	
Yes	1.00 [0.31, 3.28]	0.998	1.19 [0.19, 7.52]	0.852
**qXR score**				
≥0.50	ref		ref	
<0.50	4.97 [1.88, 13.14]	**0.001**	0.93 [0.25, 3.49]	0.917

^1^ Wald test from mixed-effect logistic regression with district as the random effect. *p*-Values < 0.05 were bolded to denote a significant difference.

## Data Availability

Study data are the property of the National TB Control Program can be furnished upon reasonable request.

## Abbreviations

The following abbreviations are used in this manuscript:

ACF	Active Case Finding
AI	Artificial Intelligence
CAD	Computer-aided Detection
CXR	Chest X-ray
HCMC	Ho Chi Minh City
NTP	National TB Control Programme
(a)OR	(adjusted) Odds Ratio
TB	Tuberculosis
Ultra	Second-generation Xpert MTB/RIF Ultra assay
Xpert	First-generation Xpert MTB/RIF assay

## References

[1] World Health Organization (2024). Global Tuberculosis Report 2024.

[2] Burke R.M., Nliwasa M., Feasey H.R.A., Chaisson L.H., Golub J.E., Naufal F., Shapiro A.E., Ruperez M., Telisinghe L., Ayles H. (2021). Community-Based Active Case-Finding Interventions for Tuberculosis: A Systematic Review. Lancet Public Health.

[3] World Health Organization (2014). Xpert MTB/RIF Implementation Manual: Technical and Operational ‘How-To’.

[4] Steingart K.R., Schiller I., Horne D.J., Pai M., Boehme C.C., Dendukuri N. (2014). Xpert ^®^ MTB/RIF Assay for Pulmonary Tuberculosis and Rifampicin Resistance in Adults. Cochrane Database Syst. Rev..

[5] Sahrin M., Rahman A., Uddin M.K.M., Kabir S.N., Kabir S., Houpt E., Banu S. (2018). Discordance in Xpert ^®^ MTB/RIF Assay Results among Low Bacterial Load Clinical Specimens in Bangladesh. Int. J. Tuberc. Lung Dis..

[6] Cepheid (2018). Xpert ® MTB/RIF Ultra Package Insert.

[7] Chakravorty S., Simmons A.M., Rowneki M., Parmar H., Cao Y., Ryan J., Banada P.P., Deshpande S., Shenai S., Gall A. (2017). The New Xpert MTB/RIF Ultra: Improving Detection of Mycobacterium Tuberculosis and Resistance to Rifampin in an Assay Suitable for Point-of-Care Testing. mBio.

[8] Dorman S.E., Schumacher S.G., Alland D., Nabeta P., Armstrong D.T., King B., Hall S.L., Chakravorty S., Cirillo D.M., Tukvadze N. (2018). Xpert MTB/RIF Ultra for Detection of Mycobacterium Tuberculosis and Rifampicin Resistance: A Prospective Multicentre Diagnostic Accuracy Study. Lancet Infect. Dis..

[9] Chilukutu L., Mwanza W., Kerkhoff A.D., Somwe P., Kagujje M., Muyoyeta M. (2022). Prevalence and Interpretation of Xpert ^®^ Ultra Trace Results among Presumptive TB Patients. Public Health Action.

[10] World Health Organization (2017). WHO Meeting Report of a Technical Expert Consultation: Non-Inferiority Analysis of Xpert MTB/RIF Ultra Compared to Xpert MTB/RIF.

[11] World Health Organization (2024). Global Tuberculosis Report 2024—Country Profile: Viet Nam.

[12] Central Coordinating Committee (2017). Proceedings on the Increase of the Protection, Care and Improvement of Population Health in the New Situation.

[13] Office of the Prime Minister (2014). Approval of the National Strategy for TB Prevention and Control until 2020 with Vision to 2030.

[14] Vietnam Ministry of Health (2020). Guidelines on the Diagnosis, Treatment and Prevention of Tuberculosis.

[15] Vietnam Ministry of Health (2024). Guidelines on the Diagnosis, Treatment and Prevention of Tuberculosis.

[16] Zifodya J.S., Kreniske J.S., Schiller I., Kohli M., Dendukuri N., Schumacher S.G., Ochodo E.A., Haraka F., Zwerling A.A., Pai M. (2021). Xpert Ultra versus Xpert MTB/RIF for Pulmonary Tuberculosis and Rifampicin Resistance in Adults with Presumptive Pulmonary Tuberculosis. Cochrane Database Syst. Rev..

[17] Saavedra B., Mambuque E., Nguenha D., Gomes N., Munguambe S., García J.I., Izco S., Acacio S., Murias-Closas A., Cossa M. (2021). Performance of Xpert MTB/RIF Ultra for Tuberculosis Diagnosis in the Context of Passive and Active Case Finding. Eur. Respir. J..

[18] Biswas S., Uddin M.K.M., Paul K.K., Ather M.F., Ahmed S., Nasrin R., Kabir S., Heysell S.K., Banu S. (2022). Xpert MTB/RIF Ultra Assay for the Detection of Mycobacterium Tuberculosis in People with Negative Conventional Xpert MTB/RIF but Chest Imaging Suggestive of Tuberculosis in Dhaka, Bangladesh. Int. J. Infect. Dis..

[19] da Silva M.P., Cassim N., Ndlovu S., Marokane P.S., Radebe M., Shapiro A., Scott L.E., Stevens W.S. (2023). More Than a Decade of GeneXpert^®^ Mycobacterium Tuberculosis/Rifampicin (Ultra) Testing in South Africa: Laboratory Insights from Twenty-Three Million Tests. Diagnostics.

[20] Iem V., Chittamany P., Suthepmany S., Siphanthong S., Siphanthong P., Somphavong S., Kontogianni K., Dodd J., Khan J.A.M., Dominguez J. (2022). Pooled Testing of Sputum with Xpert MTB/RIF and Xpert Ultra during Tuberculosis Active Case Finding Campaigns in Lao People’s Democratic Republic. BMJ Glob. Health.

[21] Vo L.N.Q., Codlin A.J., Forse R.J., Nguyen N.T., Vu T.N., Le G.T., Van Truong V., Do G.C., Dang H.M., Nguyen L.H. (2020). Evaluating the Yield of Systematic Screening for Tuberculosis among Three Priority Groups in Ho Chi Minh City, Viet Nam. Infect. Dis. Poverty.

[22] Mac T.H., Phan T.H., Nguyen V.V., Dong T.T.T., Le H.V., Nguyen Q.D., Nguyen T.D., Codlin A.J., Mai T.D.T., Forse R.J. (2020). Optimizing Active Tuberculosis Case Finding: Evaluating the Impact of Community Referral for Chest X-Ray Screening and Xpert Testing on Case Notifications in Two Cities in Viet Nam. Trop. Med. Infect. Dis..

[23] Creswell J., Vo L.N.Q., Qin Z.Z., Muyoyeta M., Tovar M., Wong E.B., Ahmed S., Vijayan S., John S., Maniar R. (2023). Early User Perspectives on Using Computer-Aided Detection Software for Interpreting Chest X-Ray Images to Enhance Access and Quality of Care for Persons with Tuberculosis. BMC Glob. Public Health.

[24] Nguyen L.H., Codlin A.J., Vo L.N.Q., Dao T., Tran D., Forse R.J., Vu T.N., Le G.T., Luu T., Do G.C. (2020). An Evaluation of Programmatic Community-Based Chest X-Ray Screening for Tuberculosis in Ho Chi Minh City, Vietnam. Trop. Med. Infect. Dis..

[25] Coleman M., Lowbridge C., du Cros P., Marais B.J. (2024). Community-Wide Active Case Finding for Tuberculosis: Time to Use the Evidence We Have. Trop. Med. Infect. Dis..

[26] Mpagama S.G., Msaji K.S., Kaswaga O., Zurba L.J., Mbelele P.M., Allwood B.W., Ngungwa B., Kisonga R.M., Lesosky M., Rylance J. (2021). The Burden and Determinants of Post-TB Lung Disease. Int. J. Tuberc. Lung Dis..

[27] World Health Organization (2011). Early Detection of Tuberculosis. An Overview of Approaches, Guidelines and Tools.

[28] Ayabina D.V., Gomes M.G.M., Nguyen N.V., Vo L., Shreshta S., Thapa A., Codlin A.J., Mishra G., Caws M. (2021). The Impact of Active Case Finding on Transmission Dynamics of Tuberculosis: A Modelling Study. PLoS ONE.

[29] Marks G.B., Nguyen N.V., Nguyen P.T.B., Nguyen T.A., Nguyen H.B., Tran K.H., Nguyen S.V., Luu K.B., Tran D.T.T., Vo Q.T.N. (2019). Community-Wide Screening for Tuberculosis in a High-Prevalence Setting. N. Engl. J. Med..

[30] Frascella B., Richards A.S., Sossen B., Emery J.C., Odone A., Law I., Onozaki I., Esmail H., Houben R.M.G.J. (2021). Subclinical Tuberculosis Disease—A Review and Analysis of Prevalence Surveys to Inform Definitions, Burden, Associations, and Screening Methodology. Clin. Infect. Dis..

[31] Falzon D., Miller C., Law I., Floyd K., Arinaminpathy N., Zignol M., Kasaeva T. (2024). Managing Tuberculosis before the Onset of Symptoms. Lancet Respir. Med..

[32] World Health Organization (2016). Chest Radiography in Tuberculosis Detection—Summary of Current WHO Recommendations and Guidance on Programmatic Approaches.

[33] Ma Z., Zhang L., Li S., Shang Y., Wang Y., Xue Z., Shu W., Sun Y., Gao X., Liu Y. (2024). Pooled Sputum Testing by Xpert^®^ MTB/RIF Ultra for Active Tuberculosis Case Finding among High-Risk Groups in a Low-Incidence Area: A Prospective Study. Infect. Dis..

[34] Codlin A.J., Vo L.N.Q., Garg T., Banu S., Ahmed S., John S., Abdulkarim S., Muyoyeta M., Sanjase N., Wingfield T. (2024). Expanding Molecular Diagnostic Coverage for Tuberculosis by Combining Computer-Aided Chest Radiography and Sputum Specimen Pooling: A Modeling Study from Four High-Burden Countries. BMC Glob. Public Health.

